# Ultrasound cystic artery velocity as a predictor for acute cholecystitis in patients presenting to the emergency department

**DOI:** 10.1007/s00261-025-05216-z

**Published:** 2025-10-13

**Authors:** Saubhagya Srivastava, Manish Dhyani, Manjiri Dighe, Tushar Kumar, David T. Fetzer, Guilherme M. Cunha, Theodore J. Dubinsky

**Affiliations:** 1 Department of Radiology, University of Washington, Seattle, USA; 2 Department of Radiology, The University of Texas Southwestern Medical Center, Dallas, USA

**Keywords:** Cystic artery velocity, Acute cholecystitis, Ultrasound, Emergency department

## Abstract

**Background:**

Acute cholecystitis (AC) is a common yet challenging diagnosis in the emergency department (ED), with diverse sonographic parameters employed to enhance diagnostic accuracy. Recently, peak systolic cystic artery velocity (CaV) ≥ 40 cm/s has been proposed as a highly specific and an independent sonographic marker for AC in the emergency setting.

**Objective:**

To evaluate the diagnostic performance of CaV for AC and to assess the diagnostic utility of additional ultrasound parameters for the diagnosis of AC in the ED.

**Methods:**

This retrospective, single-institutional study analyzed ultrasound exams from 405 patients over one year. CaV was compared in patients diagnosed with AC on surgical pathology versus controls (subjects without presumed AC), employing statistical tools for data analysis including assessment of diagnostic efficacy of sonographic markers and multivariate logistic regression to assess true sonographic predictors of AC.

**Results:**

CaV ≥ 40 cm/s demonstrated specificity and negative predictive value of 83.8% and 91.3%, respectively, but lower sensitivity and positive predictive value of 58.5% and 40.9%, respectively. The ROC curve analysis for CaV yielded an area under the curve of 0.771. On multivariate analysis several sonographic features showed significant predictors of AC in the ED, including CaV, gallbladder wall thickness, longitudinal length, presence of gallstones, and a positive sonographic Murphy’s sign.

**Conclusion:**

While CaV shows promise as a diagnostic marker, its utility should only be considered alongside other sonographic parameters due to its low independent sensitivity and positive predictive value. The sonographic diagnosis of AC in the ED continues to rely on a multiparametric approach to enhance diagnostic efficacy, ensuring more accurate and timely diagnosis of AC in the ED.

## Introduction

Acute cholecystitis (AC) is an acute inflammatory condition of the gallbladder (GB), affecting approximately 200,000 individuals per year in the United States [1]. 90–95% of AC cases are due to obstruction of the cystic duct from a gallstone (i.e., acute calculous cholecystitis) [1, 2]. While most patients with AC present to the emergency department (ED) with acute onset right upper quadrant (RUQ) pain, acute right upper abdominal or flank pain are nonspecific and a common presenting complaint in the ED, representing 5–10% of visits [3, 4], and hence, the clinical differential remains broad. Aiming to reduce ambiguity of clinical diagnosis, and considering advantages such as non-invasiveness, wide availability, and cost-effectiveness, multiple societal guidelines have established ultrasound (US) as the best first-line imaging modality for investigation of upper abdominal pain and/or suspected AC [5–7].

However, the diagnostic performance of US in AC is variable, reported to range from 27 to 93% in sensitivity and 48–99% in specificity [8–14]. The variability in ultrasound’s diagnostic performance for AC is likely due to differences in study designs, operator dependency, and patient populations. Sonographic features of AC include gallbladder distension, gallbladder wall thickening, gallstones or retained debris, pericholecystic fluid and edema. These features are best used in combination and when supported by clinical information, since individually they can be less specific and of limited use in isolation. More recently, elevated cystic artery velocity (CaV) has been proposed as a potential additional marker for AC, with recent reports suggesting ≥ 40 cm/s on spectral Doppler ultrasound to be highly specific and an independent predictor of AC in the emergency setting, with reported positive predictive values (PPV) up to 95%. While these studies stem from the theory that inflammation of the gallbladder wall in AC leads to hyperemia and a dilated cystic artery and raises velocity in the cystic artery [15, 16], limitations of these prior reports include small cohort and population bias.

In this study, we aim to further explore the diagnostic performance of CaV for AC, and secondarily, investigate the diagnostic performance of other several US parameters for the diagnosis of AC in the ED.

## Materials and methods

### Study design and patient population

This retrospective single-institution study was approved by the Institutional Review Board, with a waiver for informed consent. Patients were identified through a comprehensive review of electronic medical records (EMR) and radiology databases, targeting all right upper quadrant (RUQ) ultrasound examinations performed in the ED over the period of 1 year (January 1, 2023, to December 31, 2023). In our institution, RUQ ultrasound is the order entry used by ED physicians when acute cholecystitis is a leading differential. This list of US exams was cross-referenced with EMR records of cholecystectomies and gallbladder drainage procedures to define and categorize patients into two cohorts: a group of patients diagnosed with AC and a control group.

### Inclusion and exclusion criteria

The case cohort included adult patients (age ≥ 18 years) who underwent cholecystectomy or gallbladder drainage within 7 days of presentation to the ED and had confirmed AC via surgical pathology or documentation of turbid/dark fluid at drainage. The control group comprised two distinct but collectively analyzed subgroups:

1. *Patients with chronic cholecystitis confirmed on surgical pathology*: These cases provide a contrast by including cases characterized by chronic inflammation, providing distinction from AC cases.
2. *Patients without* AC: Patients who underwent a RUQ ultrasound and were clinically and radiologically deemed not to have AC and/or had no follow-up intervention: These cases serve as a broader negative control, encompassing patients who would not meet clinical or radiological criteria for AC when presenting to the ED.

**Fig. 1:**
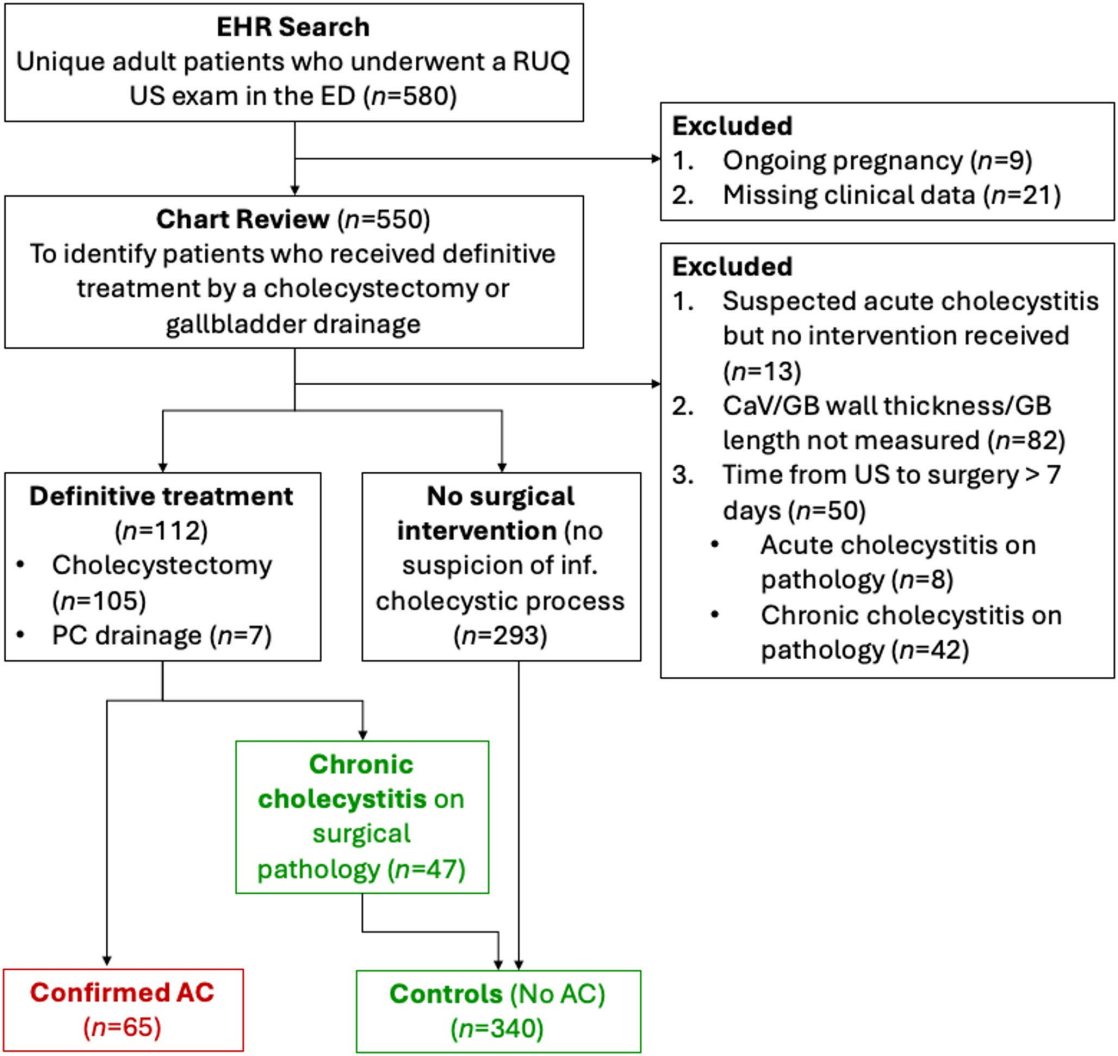
A Flowchart depicting the process of inclusion of the final sample. (*EHR* electronic health record, *RUQ* right upper quadrant, *US* ultrasound, *ED* emergency department). *CaV* cystic artery velocity, *AC* acute cholecystitis, *CC* chronic cholecystitis, *GB* gallbladder, *PC* percutaneous, *inf.* inflammatory)

Patients were excluded if they were pregnant, had incomplete US reports, or had missing clinical or sonographic data. Upon chart review, additional exclusions were applied to patients who had any level of suspicion of having AC, but did not receive intervention for any reason. This approach ensures a comprehensive evaluation by comparing AC patients against a control group that includes both chronic cholecystitis and presumed non-inflammatory cases, thereby enhancing the study’s ability to generalize the findings to a real-world clinical setting. We conducted additional analyses excluding the chronic cholecystitis subgroup from the control group to assess potential mixing bias. Figure 1 displays a flowchart depicting the inclusion of the final sample.

### Data extraction

The final US reports were extracted from PACS. Clinical, surgical, and pathology data were extracted from EMR. Clinical data included white blood cell (WBC) count, heart rate, temperature, and RUQ pain on presentation to the ED. Data were anonymized and exported for offline review. US reports were reviewed and features of AC manually extracted from the reports. Table 1 summarizes the sonographic data extracted. Continuous variables such as CaV (cm/s), gallbladder wall thickness (mm), and longitudinal gallbladder length (cm), were recorded. Categorical variables such as the presence of stones, biliary sludge, sonographic Murphy’s sign, pericholecystic fluid, and/or pericholecystic fat using binary classification (presence vs. absence) were also recorded.

**Table 1:** Summary of sonographic data manually extracted from ultrasound reports (CaV, cystic artery peak systolic velocity)

Presence/absence of:	Gallstones Biliary Sludge Pericholecystic fluid Fat stranding Sonographic Murphy’s sign
Measurements of:	Longitudinal length of the gallbladder (cm) Gallbladder wall thickness (mm) Peak systolic CaV (cm/s)

### RUQ US technique

All US examinations were performed by sonographers at our institution, CaV measurements are included in our routine institutional protocol for RUQ ultrasound performed for suspected AC and the CaV peak systolic velocity was measured in the anterior wall of the gallbladder, while avoiding the portal vein and hepatic artery. US examinations were performed on an EPIQ 7 (Philips Healthcare, Bothell WA) or LOGIQ E9 (GE HealthCare, Chicago, IL) ultrasound systems using a curved array or vector transducer in the frequency range of 1 to 6 MHz. Sagittal and transverse images and cine-loops were acquired through the gallbladder. Gallbladder wall thickness and length were measured in the long orientation. Transverse and sagittal images of the gallbladder in left lateral decubitus positions were also acquired to evaluate for mobility of stones or other intraluminal material. Color Doppler US of the gallbladder was performed for wall vascularity and to guide spectral Doppler analysis. Peak systolic velocity of the cystic artery was sampled within wall of the anterior gallbladder with angle correction performed as necessary.

### Statistical analysis

Continuous and categorical variables were compared between groups using the independent samples *t*-test and Chi-squared test, respectively. Diagnostic performance including sensitivity, specificity, positive predictive value (PPV), negative predictive value (NPV), and accuracy were evaluated for each sonographic parameter tested Univariate analysis was performed to test the association between individual US features and AC. A multivariate logistic regression was performed to assess the relation between features identified on univariate analysis and AC. A receiver operator characteristic (ROC) curve model was built for CaV and optimal cut-off threshold was identified using Youden’s index. Cut-off values at 90% sensitivity and 90% specificity were also evaluated. Sensitivity, specificity, PPV, and NPV were calculated for different cut-off points. Results were considered statistically significant at a *p*-value of < 0.05.

Statistical analysis including descriptive statistics, tests of diagnostic performance, univariate, and multivariate analysis was performed using DATAtab: Online Statistics Calculator (https://datatab.net) and EasyMedStat (version 3.38; www.easymedstat.com). ROC model was built using easyROC, a web-based tool for ROC curve analysis (ver. 1.3.1) using R language [17].

## Results

Of the total sample size of 405 patients, 112 patients received definitive treatment with either cholecystectomy or percutaneous gallbladder drainage within 7 days of the RUQ US in the ED. Of these, 65 patients had confirmed AC either on surgical pathology report (*n* = 58) or via documentation of dark/turbid fluid after percutaneous gallbladder drainage (*n* = 7). Baseline demographics and clinical parameters between the two groups are summarized in Table 2. Among clinical parameters evaluated, only white blood cell count and RUQ pain on presentation to the ED were found to be significantly different between the two study groups.

**Table 2:** Distribution of baseline demography and clinical characteristics between the two study cohorts

	AC (*n* = 65)	Controls (*n* = 340)	*p*-value
Age (years)	52 ± 16	48 ± 17	0.250
Gender (M: F)	1:1	1:1.5	0.117
WBC (x 10^6^/L)	12.5 ± 3.9	9.6 ± 5.1	< 0.001*
Elevated WBC (> 12 × 10^6^/L)	43 (66%)	100 (29%)	< 0.001*
Heart rate (bpm)	87 ± 20	85 ± 19	0.483
Tachycardia (> 90 bpm)	24 (37%)	106 (31%)	0.363
Temperature (^o^C)	36.5 ± 0.4	36.5 ± 0.6	0.318
Febrile (> 38 °C)	0 (0%)	8 (2%)	0.212
RUQ pain on presentation to ED	64 (98%)	240 (71%)	< 0.001*

Continuous variables are displayed as mean ± standard deviation and categorical variables are reported as frequency and percentage in parentheses

*AC* acute cholecystitis, *WBC* white blood cell, *bpm* beats per minute, *RUQ* right upper quadrant, *ED* emergency department

*Statistically significant

**Fig. 2:**
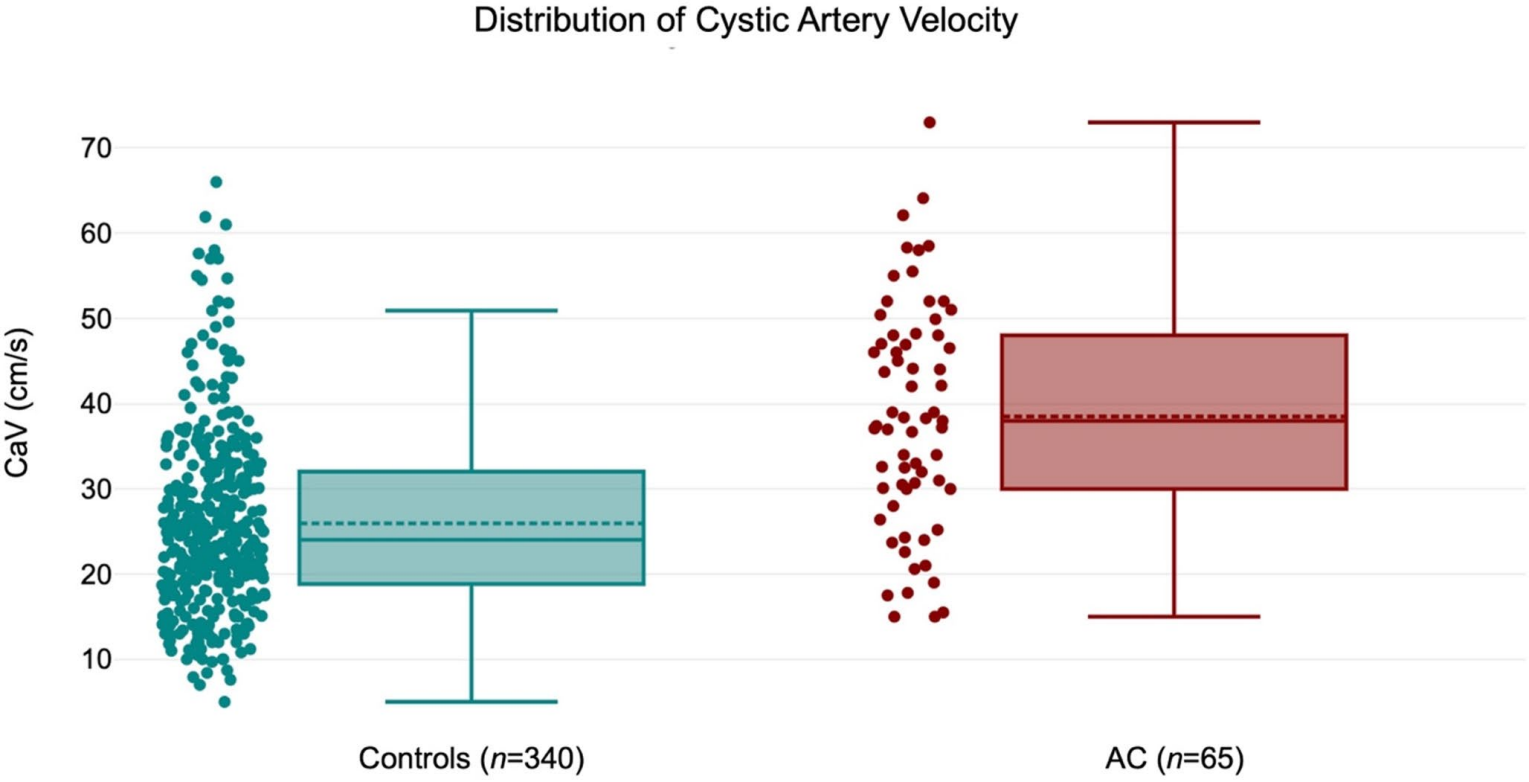
Distribution of cystic artery velocity (CaV) between the two study cohorts (AC and controls)

Among the continuous sonographic variables, CaV (cm/s), gallbladder wall thickness (mm), and length (cm) were found to be significantly different between the two cohorts (*p* = < 0.001). The AC group had significantly higher mean CaV, gallbladder wall thickness, and gallbladder length, measuring 38.5 ± 13.3 cm/s, 4.4 ± 2.7 mm, and 10.4 ± 2.0 cm, respectively, compared to the control group with mean CaV, gallbladder wall thickness, and gallbladder length of 26.0 ± 10.8 cm/s, 3.0 ± 1.8 mm, and 8.1 ± 2.1 cm, respectively (*p* < 0.001). The distribution of CaV between the two study cohorts is shown in Fig. 2. All sonographic variables except the presence of pericholecystic fat stranding (*p* = 0.320) were found to be significantly different between the two study groups (*p* = < 0.001). The distribution of sonographic features among the two study cohorts is summarized in Table 3.

**Table 3:** Distribution of sonographic features between the two study cohorts

	AC (*n* = 65)	Controls (*n* = 340)	*p*-value
CaV (cm/s)	38.5 ± 13.3	26.0 ± 10.8	< 0.001*
CaV ≥ 40 cm/s	38 (58%)	55 (16%)	< 0.001*
Gallbladder wall thickness (mm)	4.4 ± 2.7	3.0 ± 1.8	< 0.001*
Thickened gallbladder wall (≥ 3 mm)	51 (78%)	111 (33%)	< 0.001*
Gallbladder length (cm)	10.4 ± 2.0	8.1 ± 2.1	< 0.001*
Gallbladder length ≥ 8 cm	57 (88%)	171 (50%)	< 0.001*
Presence of stones	61 (94%)	159 (47%)	< 0.001*
Biliary sludge	30 (46%)	73 (21%)	< 0.001*
Pericholecystic fluid	27 (41%)	50 (15%)	< 0.001*
Pericholecystic fat stranding	5 (8%)	16 (5%)	0.320
Positive sonographic Murphy’s sign	24 (37%)	25 (7%)	< 0.001*

Continuous variables are displayed as mean ± standard deviation and categorical variables are reported as frequency and percentage in parentheses. *AC* acute cholecystitis, *CaV* cystic artery velocity

*Statistically significant

**Fig. 3:**
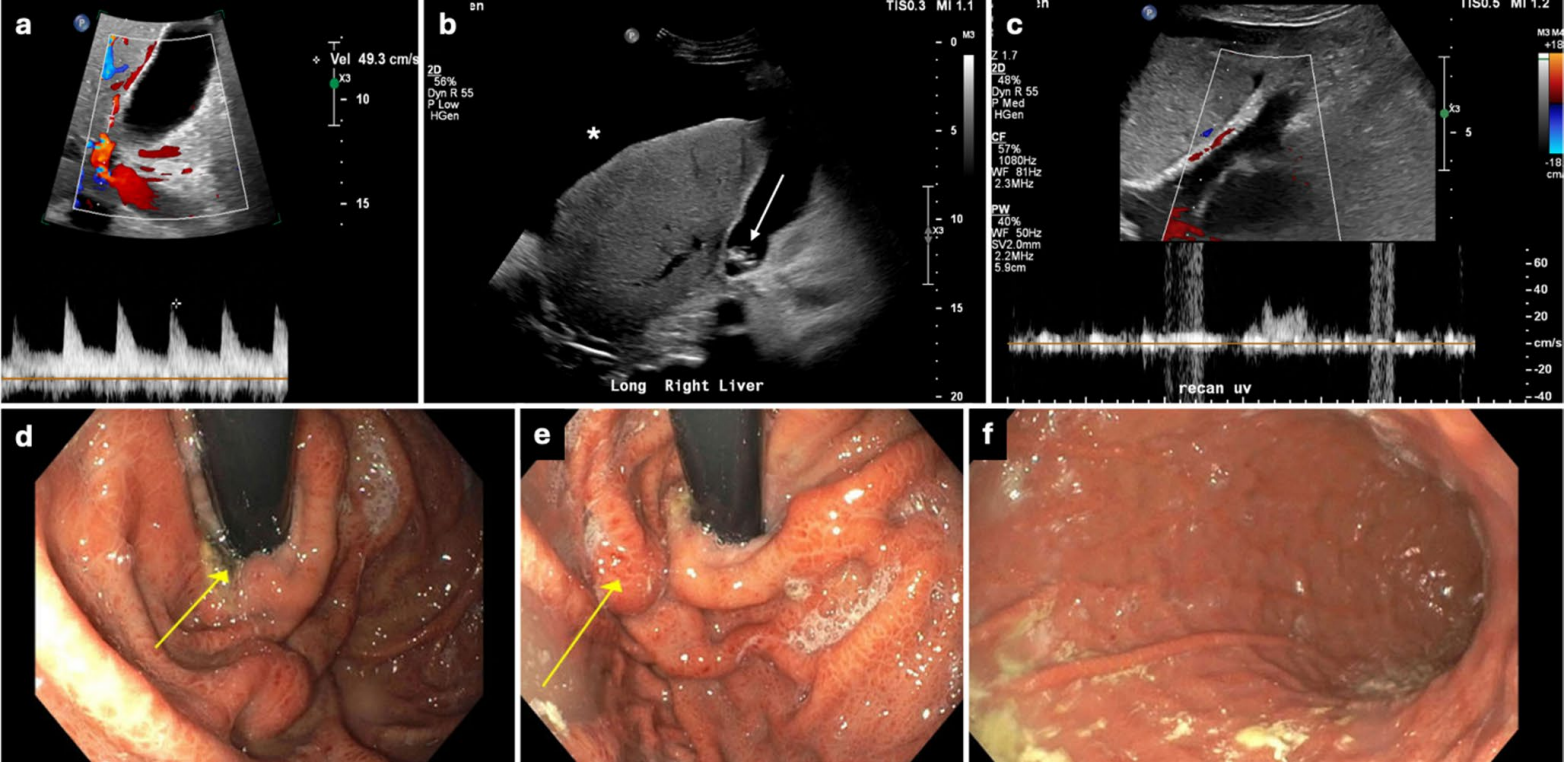
A 42-year-old male with history of alcohol consumption and gallstones presented to the ED with RUQ pain and hematemesis. RUQ US showed an elevated CaV of 49 cm/s (**a**), echogenic gallstones (arrow in **b**), cirrhotic liver morphology, and ascites (asterisk in **b**). There was also a patent paraumbilical vein (**c**). Esophagogastroduodenoscopy (EGD) revealed esophageal ulcers (**d**), gastroesophageal varices (**e**), and mosaic-like gastric mucosa (**f**), typical of hypertensive gastropathy

**Fig. 4:**
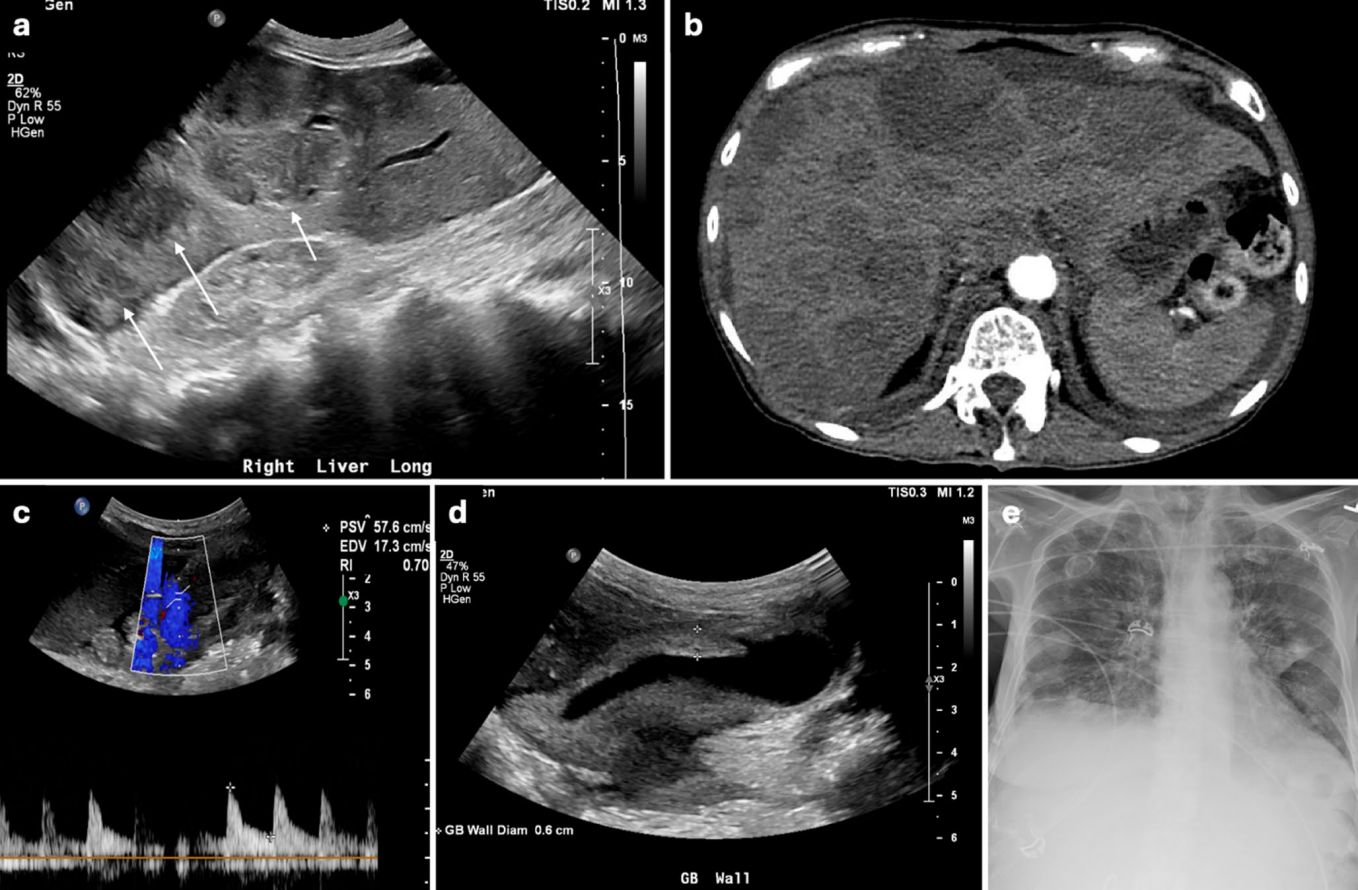
A 78-year-old female with known endometrial carcinoma presented to the ED with shortness of breath. A RUQ US showed multiple hepatic metastases (arrows in **a**), similar to CECT findings a few days earlier (**b**), an elevated CaV of 58 cm/s (**c**), and a circumferentially thickened gallbladder (calipers in **d**), likely reactive given adjacent hepatic disease and absence of physical exam findings of cholecystitis. Bilateral pleural effusions were also noted (**e**)

**Fig. 5:**
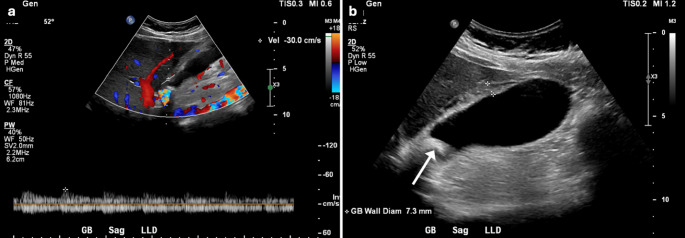
A 46-year-old female with history of large gallstones presented to the ED with RUQ abdominal pain. RUQ US showed normal CaV of 30 cm/s (**a**), a distended gallbladder with significantly thickened call (calipers in **b**), and a large gallstone lodged in the gallbladder neck (arrow in **b**). Same-day cholecystectomy confirmed AC

**Fig. 6:**
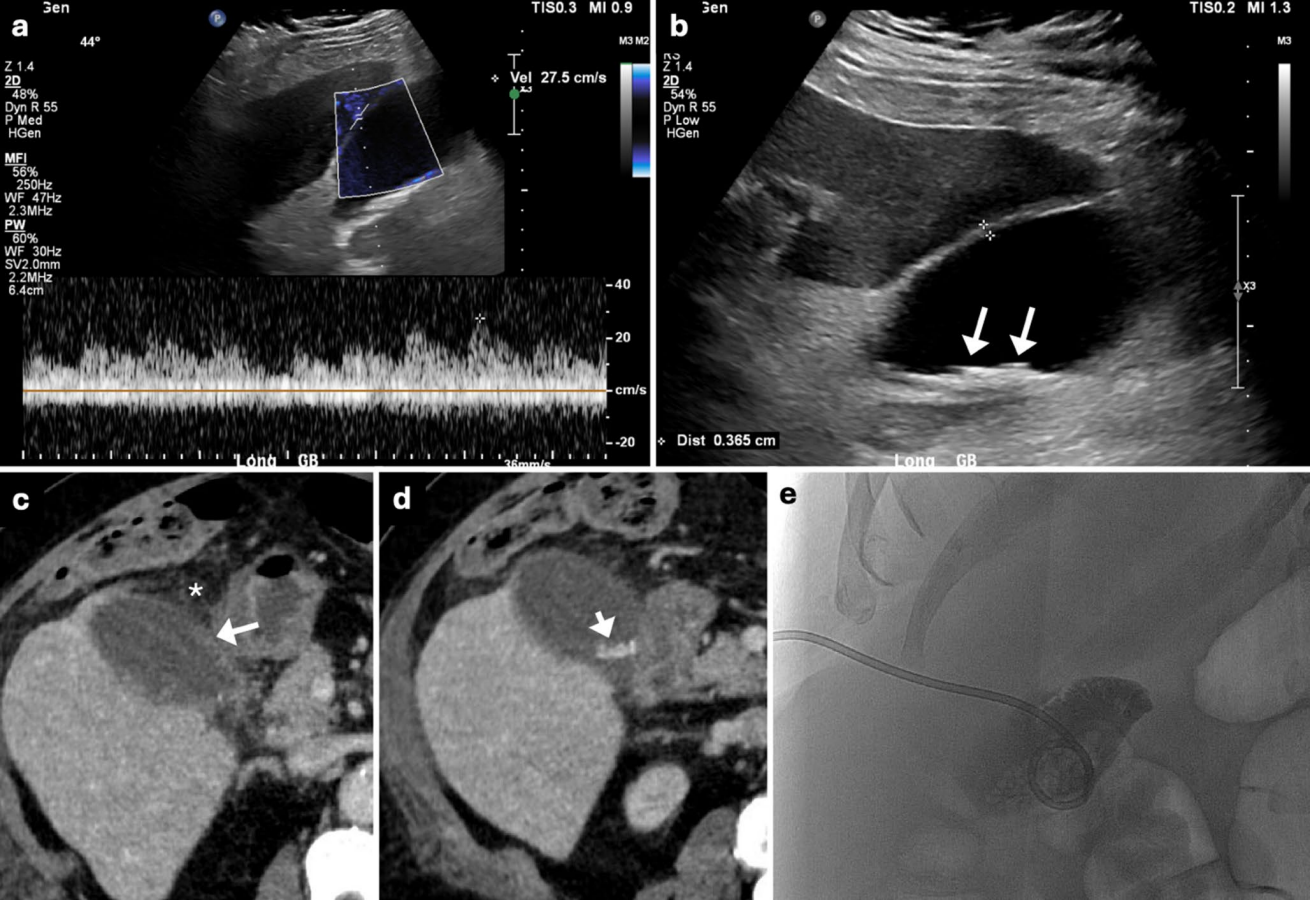
A 58-year-old male presented to the ED with diffuse abdominal pain. RUQ US showed normal CaV of 28 cm/s (**a**), a distended gallbladder (**b**), echogenic gallstones (arrows in b), and a thickened wall (calipers in **b**). Same-day CECT also showed a thickened wall (arrow in **c**) with surrounding pericholecystic fluid, fat stranding (asterisk in **c**), and hyperattenuating gallstones (arrow in **d**). Fluid culture after percutaneous gallbladder drainage (**e**) confirmed AC

CaV ≥ 40 cm/s had a sensitivity, specificity, positive predictive value (PPV), negative predictive value (NPV), and diagnostic accuracy of 58.5%, 83.8%, 40.9%, 91.3% and 79.7%, respectively. All sonographic features had high NPVs ranging from 84.4% to 97.8%. Presence of gallstones had the highest sensitivity (93.8%) compared to other sonographic parameters in evaluating AC. Table 4 summarizes the tests of diagnostic efficacy across all sonographic parameters tested. In our study’s database, 93 subjects had an elevated CaV ≥ 40 cm/s. Of these, 55 (59%) were classified as false positives. Similarly, of 312 subjects with normal CaV (< 40 cm/s), 27 (9%) were classified as false negatives. Figures 3 and 4 display representative examples of false positive cases with diagnoses of alcoholic cirrhosis with gastrointestinal hemorrhage and acute pericardial effusion, respectively. Figures 5 and 6 display representative examples of false negative cases. This overlap is also appreciated in Fig. 2, wherein several subjects in the control group are seen with elevated velocities in the cystic artery.

**Table 4:** Diagnostic performance of different sonographic features in the diagnosis of AC compared to controls

	Sensitivity (%)	Specificity (%)	PPV (%)	NPV (%)	Accuracy (%)
CaV ≥ 40 cm/s	58.5 [45.7, 70.6]	83.8 [79.5, 87.6]	40.9 [33.5, 48.7]	91.3 [88.7, 93.4]	79.7 [75.5, 83.6]
Gallbladder wall thickness ≥ 3 mm	78.5 [66.5, 87.7]	67.4 [62.1, 72.3]	31.5 [27.4, 35.9]	94.2 [91.1, 96.3]	69.1 [64.4, 73.6]
Gallbladder length ≥ 8 cm	87.7 [77.2, 94.5]	49.7 [44.3, 55.2]	25.0 [22.5, 27.7]	95.5 [91.6, 97.6]	55.8 [50.8, 60.7]
Presence of gallstones	93.8 [85.0, 98.3]	53.2 [47.8, 58.6]	27.7 [25.2, 30.4]	97.8 [94.6, 99.2]	59.8 [54.8, 64.6]
Presence of biliary sludge	46.2 [33.7, 59.0]	78.5 [73.8, 82.8]	29.1 [22.7, 36.4]	88.4 [85.8, 90.1]	73.3 [68.7, 77.6]
Pericholecystic fluid	41.5 [29.4, 54.4]	85.3 [81.1, 88.9]	35.1 [26.9, 44.3]	88.4 [86.1, 90.4]	78.3 [73.9, 82.2]
Positive sonographic Murphy’s sign	36.9 [25.3, 49.8]	92.7 [89.3, 95.2]	49.0 [37.0, 61.1]	88.5 [86.4, 90.3]	83.7 [79.7, 87.2]

Values of sensitivity, specificity, PPV, and NPV are reported in percentages with 95% confidence intervals in [square brackets] (*PPV* positive predictive value, *NPV* negative predictive value, *CaV* cystic artery velocity, *PC* pericholecystic)

*Statistically significant

Repeating the diagnostic performance analysis after excluding the chronic cholecystitis subgroup (*n* = 47) from controls did not change CaV performance estimates. After exclusion of the chronic cholecystitis group, CaV ≥ 40 cm/s had a sensitivity, specificity, PPV, and NPV of 58.5%, 85.3%, 40.9%, and 90.3%, respectively. Similarly, the AUC for CaV was 0.778 after exclusion of the chronic cholecystitis group, virtually unchanged from the original control group. This sensitivity analysis indicates that combining the control subgroups (chronic cholecystitis and non-AC groups) did *not* meaningfully bias our results.

Univariate analysis of sonographic features revealed that an elevated CaV ≥ 40 cm/s (p = < 0.001), thickened gallbladder wall ≥ 3 mm (p = < 0.001), gallbladder length ≥ 8 cm (p = < 0.001), presence of gallstones (p = < 0.001), biliary sludge (p = < 0.001), pericholecystic fluid (p = < 0.001), and positive sonographic Murphy’s sign (p = < 0.001) were statistically significant predictors of AC. Table 5 shows a summary of the univariate analysis of the sonographic features. On multivariate analysis of above-mentioned significant variables, an elevated CaV ≥ 40 cm/s (*p* = 0.002), thickened gallbladder wall ≥ 3 mm (p = < 0.001), gallbladder length ≥ 8 cm (*p* = 0.008), presence of gallstones (p = < 0.001), and a positive sonographic Murphy’s sign (*p* = 0.033) remained statistically significant predictors of AC. Tables 5 and 6 show a summary of the univariate and multivariate analysis of sonographic variables tested, respectively.

**Table 5:** Results of univariate analysis of sonographic features in relation to the diagnosis AC versus controls

Sonographic Feature	Odds Ratio (OR)	*p*-value
CaV ≥ 40 cm/s	7.29 [4.12; 12.92]	< 0.001*
Gallbladder wall thickness (≥ 3 mm)	7.52 [3.99; 14.16]	< 0.001*
Gallbladder length ≥ 8 cm	7.04 [3.26; 15.21]	< 0.001*
Presence of gallstones	17.36 [6.17; 48.81]	< 0.001*
Presence of biliary sludge	3.14 [1.8; 5.45]	< 0.001*
Pericholecystic fluid	4.12 [2.31; 7.34]	< 0.001*
Pericholecystic fat	1.69 [0.6; 4.78]	0.356
Positive sonographic Murphy sign	7.38 [3.86; 14.1]	< 0.001*

*Statistically significant

**Table 6:** Results of multivariate analysis of sonographic features in relation to diagnosis of AC versus controls

Sonographic Feature	Odds Ratio (OR)	*p*-value
CaV ≥ 40 cm/s	2.86 [1.45, 5.64]	0.002*
Gallbladder wall thickness (≥ 3 mm)	3.84 [1.85, 7.96]	< 0.001*
Gallbladder length ≥ 8 cm	3.25 [1.36, 7.78]	0.008*
Presence of gallstones	6.96 [2.32, 20.91]	< 0.001*
Presence of biliary sludge	1.79 [0.899, 3.58]	0.098
Pericholecystic fluid	1.33 [0.645, 2.76]	0.437
Positive sonographic Murphy’s sign	2.39 [1.07, 5.34]	0.033*

*Statistically significant

**Fig. 7:**
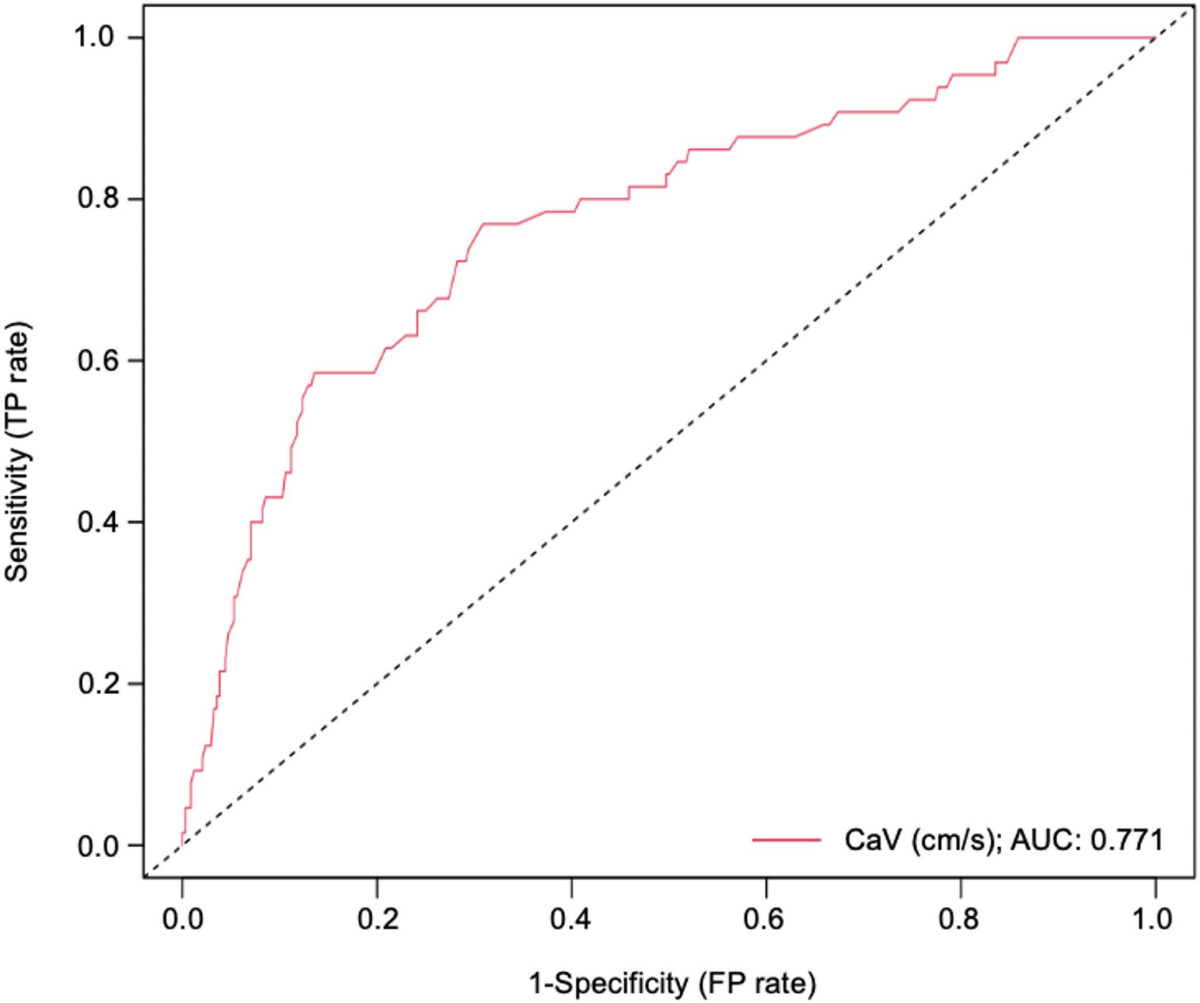
Receiver operator characteristic (ROC) curve for cystic artery velocity (CaV). The area under the curve (AUC) was 0.771 (95% CI: 0.706–0.836). (*TP* true positive, *FP* false positive)

A receiver operator characteristic (ROC) curve was built for CaV (cm/s) (Fig. 7). The area under the curve (AUC) for CaV was 0.771. Using Youden index analysis, the optimal cut-offs for CaV was ≥ 30 cm/s. Table 7 shows a summary of tests of diagnostic efficacy (sensitivity, specificity, PPV, and NPV) for CaVat different cut-off points, including optimal cut-off, cut-off at 90% sensitivity, and cut-off at 90% specificity.

**Table 7:** Summary of analysis of sensitivity, specificity, PPV, and NPV for cystic artery velocity (CaV) at optimal, 90% sensitivity, and 90% specificity cut-off thresholds

Cut-off threshold	Sensitivity (%)	Specificity (%)	PPV (%)	NPV (%)
Optimal cut-off				
30 cm/s	76.9 [64.8, 86.5]	69.1 [63.9, 74.0]	32.3 [27.4, 47.7]	94.0 [89.6, 95.2]
Cut-off at ~ 90% sensitivity				
21 cm/s	89.2 [79.1, 95.6]	34.1 [29.1, 39.4]	20.6 [17.0, 40.2]	94.3 [88.3, 95.4]
Cut-off at ~ 90% specificity				
39 cm/s	43.1 [30.8, 56.0]	90.0 [86.3, 93.0]	45.2 [36.6, 58.0]	89.2 [83.0, 92.4]

## Discussion

This study further validates the high negative predictive value of CaV alongside other sonographic markers for diagnosing AC in the ED. In this study, when we reviewed the utility of peak systolic CaV (cm/s) undergoing a right upper quadrant ultrasound in the emergency department, we found that the sensitivity and PPV for CaV was 58.5% and 40.9%, respectively, using ≥ 40 cm/s as the cut-off value. While these findings are contrary to previously published manuscripts, the specificity of CaV in our dataset remains high (83.8%); consistent and in agreement with previous publications [15, 18]. These findings challenge the prevailing assumption of CaV’s standalone reliability in diagnosing AC, underscoring the necessity of integrating this measurement with additional sonographic markers to enhance diagnostic precision. Ultimately, this study concludes that CaV, though useful, cannot singularly fulfill the diagnostic needs for AC without the support of a multiparametric sonographic approach. In our cohort, although CaV contributed meaningfully when interpreted alongside other sonographic markers, its standalone performance was insufficient for definitive rule-in of AC. We therefore view CaV as an adjunct for equivocal cases (e.g., discordant or borderline sonographic findings, analgesia-blunted Murphy’s sign, or equivocal secondary sonographic signs), rather than a universal screen. In practice, a tiered approach may be reasonable: CaV ≥ 40 cm/s strongly supports AC *when other sonographic findings are also present*; 30–39 cm/s constitutes a gray zone that warrants closer investigation with clinical/lab/sonographic data; and < 30 cm/s reduces the likelihood of AC (high NPV), especially when additional sonographic signs are absent.

The low sensitivity and PPV implies that many patients with AC may be overlooked if CaV is used in isolation. Moreover, the optimal cut-off identified through ROC curve analysis in our study was lower at ≥ 30 cm/s. However, individual cut-offs are dependent on prevalence of disease in individual study cohorts, perhaps explaining the discrepancy between the cut-offs in our study and prior literature [15]. The adjustment in cut-off level to ≥ 30 cm/s in our study improved the sensitivity to 77% while maintaining a specificity of 70%, but the PPV remained low (32%). Additionally, multivariate analysis revealed that the integration of other sonographic significantly enhanced the diagnostic accuracy. Traditionally used sonographic markers such as presence of gallstones, gallbladder wall thickening, gallbladder length ≥ 8 cm, and a positive sonographic Murphy’s sign were shown to strongly correlate with AC when combined with an elevated CaV, supporting a more nuanced multiparametric approach [6, 7, 18]. Traditionally used sonographic Murphy’s sign had the highest specificity (92.7%) and diagnostic accuracy (83.7%) in our dataset, which is similar to recently published data by Patel et al. [19], where the authors concluded that sonographic Murphy’s sign combined with age, sex, and WBC count stratifies ED patients into AC risk groups [19]. Our study revealed that most sonographic markers displayed low PPVs, ranging from 23.8 to 49.0%. While sonographic Murphy’s sign is traditionally associated with a high PPV, the low PPV in this cohort could be secondary to ingestion of analgesics, presence of comorbidities that can blunt pain response (e.g., diabetes), and inherent subjectivity of the exam [18, 20]. In contrast, the NPVs for all evaluated sonographic signs were consistently high, ranging from 84.4 to 97.8%. These findings suggest that while the absence of specific sonographic signs is reliable for ruling out AC, the presence of any single sign should not clinically dictate a definitive diagnosis of AC. Furthermore, the relationship between disease prevalence and predictive values is highlighted [21]; with a prevalence of 19% in our study, the PPV is naturally limited while the NPV remains robust. These findings underscore the need for a comprehensive diagnostic approach, integrating multiple sonographic findings to enhance accuracy in the clinical assessment of AC.

Our findings contrast with those from Perez et al. [15], where the authors found CaV to be a standalone predictor of AC in the ED. Instead, our data supports that no single sonographic feature can reliably diagnose AC independently. As in our multivariate analysis of sonographic parameters, prior studies have also found the presence of multiple sonographic markers to increase diagnostic accuracy for AC [11, 22, 23]. A 1992 study that evaluated gallbladder arteriograms proved that gallstone-related cholecystitis showed a higher degree of arterial dilation compared to acalculous cholecystitis [24]. Notably, although gallbladder wall hyperemia leading to elevated CaV may be a feature of AC, it is not specific or predictive and may be present in other diseases of the gallbladder including chronic cholecystitis, gallbladder carcinoma, adenomyomatosis, and even extra-cholecystic inflammation [25]. The highest odds ratio and significance was seen with the presence of gallstones, which is in agreement with the pathophysiology of AC, most commonly beginning with the impaction of gallstone in the cystic duct leading to mechanical obstruction to the flow of bile and subsequent chemical irritation of the gallbladder [26]. Gallbladder distension, traditionally defined as longitudinal and transverse dimensions ≥ 8 and ≥ 4 cm, respectively, is a common feature in AC [6]. A recent study by Shaish et al. [27] found 100% sensitivity in ruling out AC when the gallbladder was under-distended (defined as transverse dimension of < 2.2 cm) [27].

The diagnosis of AC using sonographic markers continues to rely on a multi-parametric approach. As seen in our results, no individual sonographic sign had a high PPV, although several were statistically significant on multivariate analysis. Implementing these findings into clinical practice could enhance diagnostic strategies used in emergency departments towards AC. As RUQ ultrasound is the first-line diagnostic modality for someone presenting to the ED with RUQ pain, adopting a multi-parametric diagnostic criterion can improve the accuracy of diagnoses and ensure that patients receive timely and appropriate intervention. Such an approach could help minimize unnecessary interventions and optimize utilization of healthcare resources, leading to improved patient outcomes in the acute setting. It should be remembered that Doppler angle effects and sampling technique can degrade reliability; CaV can be incorporated as an adjunct (*not* a standalone predictor) to increase confidence in indeterminate studies using a tiered approach, while maintaining a multiparametric decision pathway.

### Limitations and future directions

We acknowledge that our study had several limitations. First and foremost, by nature of this being a retrospective study, inherent limitations of a retrospective data collection exist. Additionally, velocities measured in this retrospective study were not angle-corrected for comparison purposes. CaV Doppler estimation depends on the insonation angle, and small angle deviations can produce differences in the measured velocity [28]. Although our institutional protocol permits angle correction, we did not standardize angle targets due to the retrospective nature of this study. Prospective protocols may enforce angles ≤ 60^o^, which will aid in determining the true characteristics of CaV under standardized acquisition. While ultrasound reports were based on interpretation at a tertiary care center, data collection was based on these diverse reads, and no additional image review was performed by dedicated readers, subjecting the data to non-conformity, and thus no inter- or intra-observer agreements could be calculated. By extension of being a retrospective review of ultrasound reports, we did not collect or measure parameters that were not clinically reported such as the transverse dimension of the gallbladder. In future studies, we aim to study the added value of transverse dimension of the gallbladder in association with AC. In addition, by nature of extracting surgical pathology data, the final diagnosis of patients who had a RUQ US was not known in all cases (e.g., patients who did not receive a definitive intervention as they were clinically presumed to not have an ongoing cholecystic process). Furthermore, our reason for selecting all RUQ US ultrasounds instead of only exams that stated the indication as RUQ pain was to negate potential bias that may arise from reason for exam given by the ED provider.

## Conclusion

The diagnosis of AC by ultrasound in patients having a RUQ US in the ED should rely on a multiparametric approach rather than a single individual marker such as CaV due to their limited sensitivity and positive predictive values when used in isolation.

## Data Availability

No datasets were generated or analysed during the current study.
